# NMR-based metabolomics profile during a soccer season of a sub-elite soccer team

**DOI:** 10.1007/s11306-026-02452-2

**Published:** 2026-05-24

**Authors:** Alisson Henrique Marinho, Filipe Antonio de Barros Sousa, Pedro Balikian Junior, Edson de Souza Bento, Thiago de Mendonça Aquino, Victor Amorim Farias Andrade-Souza, Edmilson Rodrigues da Rocha-Junior, Alessandre Crispim, Gustavo Gomes de Araujo

**Affiliations:** 1 Laboratory of Applied Sports Science, Institute of Physical Education and Sport, Federal University of Alagoas, Avenue Lourival Melo Mota, s/n, Maceió, Alagoas 57072-900 Brazil; 2 Laboratory of Nuclear Magnetic Resonance, Chemistry and Biotechnology Institute, Federal University of Alagoas, Maceió, Brazil; 3 Human and Exercise Physiology Study and Research Group, Federal Institute of Alagoas, Maragogi, Alagoas Brazil

**Keywords:** Metabolomics, Football, Exercise biochemistry, Metabolism, Sportomics

## Abstract

**Purpose:**

This study aimed to characterize metabolomic profiling, match performance, and GPS-derived variables across the competitive season of a sub-elite soccer team.

**Methods:**

Performance variables were recorded by GPS during matches. Urine samples were collected pre-match across 20 games. For integrative analysis, only players participating ≥ 25 min per match were included (*n* = 13 matches). Spectra were pre-processed using TopSpin^®^3.2, and metabolites were identified using Chenomx^®^.

**Results:**

Heatmap analysis revealed two main metabolite clusters (C): C1 (choline to citrate – C1) and C2 (dimethylamine to trans-aconitate – C2). C1 showed lower concentrations at the beginning of the season with a progressive increase toward the end, whereas C2 displayed the opposite trend. A sub-cluster within C2 (trimethylamine to trans-aconitate) exhibited a three-phase pattern, decreasing mid-season and increasing thereafter. PCA and OPLS-DA demonstrated clear separation between early- and late-season matches, indicating a shift in the metabolomic profile. Distance covered across speed zones was higher in C1 and lower in C2. Leucine, guanidinoacetate, creatinine, and 3-hydroxyisovalerate were inversely associated with distance metrics, linking elevated protein catabolism markers to reduced performance. Glycerophosphocholine was also inversely correlated with most distance variables of GPS metrics, suggesting reduced muscle integrity. In contrast, glucose was positively associated with player load, accelerations, decelerations, and impacts, while citrate correlated with high-speed running and maximum velocity.

**Conclusions:**

Seasonal metabolomic shifts are associated with external load and performance. Markers of protein catabolism relate to reduced output, whereas energy-related metabolites support high-intensity actions, highlighting metabolomics as a complementary tool for monitoring recovery and performance in soccer.

**Supplementary Information:**

The online version contains supplementary material available at 10.1007/s11306-026-02452-2.

## Introduction

During soccer matches, the players are required to perform several high- and low-intensity movements (e.g., jumping, running, change of directions, acceleration, and decelerations) (StØlen et al., 2005). Generally, the athlete participates in soccer matches twice to three times per week, separated by two to three days (Nédélec et al., 2012). The frequent execution of these movements triggers exacerbated metabolic perturbations, which are not always fully recovered between games and may result in cumulative fatigue (Silva et al., 2018). Understanding metabolic demand throughout a soccer season has been extensively investigated to reduce the probability of development of excessive skeletal muscle damage and enhance recovery (Nédélec et al. 2012; Thorpe and Sunderland 2012) hence, to guarantee performance maintenance. Among the biomarkers, lactate, urea, creatine kinase, and hormonal markers are the most commonly used to assess internal training load (Nédélec et al., 2012). However, although these metabolic markers provide valuable insights into internal load and recovery status, their interpretation in soccer is limited by high inter-individual variability, low specificity, and the strong influence of contextual and external factors, thereby reinforcing the need for a more integrated monitoring approach.

Monitoring training and match intensity is a fundamental practice in professional soccer, aiming to optimize physiological adaptations and enhance performance (Djaoui et al., 2017). Training load can be broadly classified into external and internal components. External load refers to the physical demands imposed during training and matches, typically quantified through variables such as total distance covered, accelerations, and duration. In contrast, internal load reflects the individual physiological and biological responses to these demands, including measures such as heart rate, oxygen consumption, and perceived exertion (Impellizzeri et al., 2019). In professional settings, external load is commonly monitored using electronic performance tracking systems (EPTS), particularly global positioning systems (GPS), which enable detailed quantification of time–motion characteristics, high-speed running, player load, and accelerations/decelerations (Akenhead & Nassis, 2016; Ravé et al., 2020). These metrics provide valuable insights into the physical demands of the sport, which is characterized by intermittent high-intensity efforts superimposed on predominantly low-intensity activity (Bradley et al., 2009).

Despite the widespread use of GPS-derived metrics, external load alone does not fully capture the physiological stress imposed on athletes. Similar external demands can elicit markedly different internal responses depending on individual factors such as fitness level, recovery status, nutrition, and psychological condition (Coutts et al., 2018). Therefore, the assessment of internal load is essential to understand how athletes respond to training stimuli and to prevent maladaptive outcomes such as overtraining. Traditional approaches include heart rate-based methods, lactate measurements, and perceived exertion scales (Djaoui et al., 2017), while emerging techniques such as metabolomics offer additional insights into the metabolic processes underlying training adaptation(Tindaro Bongiovanni et al., 2022).

Metabolomics is a recent approach to soccer, and has been used to measure metabolic responses to exercise using human biofluids (e.g., blood, urine, saliva, cerebrospinal fluid, and feces) analysis (Bongiovanni, Dessì, et al. 2019). There are already some systematic reviews and meta-analyses showing that using metabolomics enables detection a broad number of metabolites associated to pathways of human energy systems, skeletal muscle damage, and oxidative and anti-inflammatory processes during exercise (Sakaguchi et al. 2019a, 2019b; Schranner et al. 2020). Therefore, the metabolomics approach might be an exciting tool to assess several metabolic pathway interconnections during exercise performance from an integrated biochemical standpoint.

Although metabolomic profiling has been increasingly investigated across different exercise modalities (T. Bongiovanni, Pintus, et al. 2019; Sakaguchi et al. 2019a, 2019b; Schranner et al. 2020), studies in soccer remain limited (Marinho et al. 2022; Pintus et al. 2021; Quintas et al. 2020). Existing research has primarily focused on acute responses to matches, demonstrating significant post-game metabolic alterations (Marinho et al. 2022; Ra et al. 2014). However, investigations examining metabolomic changes across an entire season are scarce, with fewer studies addressing this approach (Pintus et al. 2021; Quintas et al. 2020). These studies either focused on pre-season responses in professional players or longitudinal observations in youth athletes, leaving a gap in understanding match-to-match metabolic fluctuations in professional soccer. Therefore, assessing metabolomic profiles prior to each match may provide valuable insights into players’ physiological readiness and help explain performance variations throughout the season.

Thus, the present study aimed to characterize metabolomic profiling changes throughout the season using a metabolomics approach, connecting them to match outcomes and GPS-derived performance variables of exercise performance.

## Materials and methods

### Participants

The entire sub-elite male professional soccer squad (*n* = 44) of a Brazilian club (age: 26 ± 4 years; height: 180.5 ± 6.9 cm; body mass: 78.4 ± 7.1 kg; body mass index: 24.02 ± 1.3; % fat mass Pollock: 8.1 ± 1.9; % fat mass Faullkner: 11.4 ± 1.4) was recruited and included in the analysis of metabolomics along the season (20 cross-sectional moments), excluding the goalkeepers. This club played in the second division of the Brazilian championship. All participants were professional athletes enrolled in the team disputing the national Brazilian championship. Among the squad, for GPS analysis and correlation to metabolomics, only players that played at least 25 min in a given match were included to analyze that specific match (total of 13 matches). This resulted in 29 athletes being included in the comparison between metabolomics and GPS analyses (*n* ≥ 10 matches = 7; *n* ≥ 5 matches = 14). All athletes were informed about all possible discomforts, risks, and benefits related to the study before signing a written informed consent form of the study. All participants were injury-free in the last three months, not smokers, and without pathology associated with the cardiovascular system. All the players were instructed not to drink alcohol or ergogenic substances, not practice vigorous physical activity during the previous 24 h before the matches and to maintain their dietary habits, including hydration. During the competitive season, pre-match routines were standardized according to match location. For home matches, players stayed at the club’s accommodation on the day preceding the game. For away matches, players traveled two days prior to the match and performed a training session in the host city on the day before the game. In both conditions, dietary intake was controlled and standardized by the club’s nutritionist for all players. Additionally, players were instructed to maintain a minimum habitual sleep duration of 8–9 h. Training load in the 24 h prior to the match was standardized, with the primary objective of implementing tactical adjustments; sessions consisted of low-intensity activities, with total distance covered not exceeding 3 km, in order to optimize recovery status before competition. According to the Helsinki declaration, this study was carried out and was approved by the local Research Ethics Committee (29269020.8.0000.5013).

### Experimental design

For the present study, athletes were assessed throughout the championship. All data regarding height, body mass, body mass index and % of fat mass was measured through the battery test carried out before the season beginning (i.e., pre-season record) or the battery test for new players integrated into the team during the season. Body fat percentage was estimated using the validated Pollock and Faulkner skinfold protocols (Jackson & Pollock, 1978; Faulkner, 1968). For participants, the 7-site method (chest, mid-axillary, triceps, subscapular, abdomen, suprailiac, and thigh) and 4-site method (triceps, suprailiac, subscapular and thigh) were used, following standardized anatomical landmarks. The skinfold thickness was measured in triplicate using calipers, and body density was calculated using gender-specific equations (Jackson & Pollock, 1978), with subsequent conversion to body fat percentage via the Siri (1961) equation.

During the season, the urine fluids (25 mL) were collected preferably before every soccer match to represent metabolomic profiling preceding the game. Of a total of 20 cross-sectional moments throughout the season, thirteen were collected before an official match and used to compare metabolomics and GPS derived variables, while the other seven were collected at moments at the request of the coaching team. Match outcomes (win, draw, or loss) corresponding to the closest fixture to each urine sampling time point were aligned with the respective metabolomic profiles to contextualize the competitive context experienced by the squad. Metabolomic profiling was performed in all athletes of the squad (*n* = 44), excluding the goalkeepers, to assess their readiness to perform throughout the season, considering all of them were enrolled in the same training regimen, including friendly matches for the ones not listed for the official games. GPS analyses and match success were carried out only for the players that participated in the official games and played for at least 25 min, to ensure performance analysis would come from a match valid for the championship. All soccer matches were performed according to the Federation International Football Association (FIFA) rulebook. The athletes were asked to provide 25 mL of their first urine of the day, collected in an aseptic container. All athletes were fully advised to follow procedures of urine sample collection: (1) wash their hands; (2) retract the penis foreskin; (3) wash the penis with soap and water; (4) dry the penis; (5) reject the first urine jet of the day to avoid accumulated residues generated during the sleep period; (6) urinate 25 mL in an aseptic container with sodium azide; (7) close the collection bottle and freeze. These procedures were carried out during every urine collection throughout the season. Urine was selected as the biofluid based on the nature and objectives of the study. It represents a non-invasive, practical, and highly suitable matrix for metabolomics investigations, particularly in sports science contexts. Also, urine provides a robust representation of systemic metabolic changes and offers broad metabolite coverage.

### Metabolomics analysis

#### Sample preparation

The urine samples were taken to the Chemistry Institute for posterior analysis. Of the 25 mL collected after the match, 1.5 mL aliquots were removed and transferred, individually, to Eppendorf tubes. After this, the samples were centrifuged at 14,000 rpm (Hettich Zentrifugen, ROTANTA 460R) for 15 min. The resulting supernatant was carefully collected and stored at − 80 °C until further analysis. Prior to analysis, samples were centrifuged again at 14,000 rpm for 15 min to remove any remaining insoluble debris. Subsequently, 500 µL of the supernatant from each sample was transferred into individual 5 mm NMR tubes. To each sample, 200 µL of a buffer solution (sodium phosphate buffer, pH 7.4) prepared in 100% D₂O and containing 1 mM TSP was added for deuterium lock signal and chemical shift referencing.

#### Sample analysis and identification of metabolites

The NMR experiments were conducted in a BRUKER 600 MHz spectrometer (AVANCE III) equipped with a 5 mm PABBO probe at 300 K. All ¹H NMR spectra were acquired using the NOESY-presaturation pulse sequence (noesygppr1d) to suppress the water signal For all samples, the following acquisition parameters were used: 128 transients, 64 K data points, a spectral width of 20 ppm, relaxation delay (D1) of 4.00 s, mixing time (D8) of 0.09 s, and an acquisition time of 2.72 s. All spectra were pre-processed using TopSpin^®^ 3.2 software, including phase correction and chemical shift calibration referenced to the TSP signal at 0.00 ppm. Data processing was subsequently performed in MATLAB^®^ (MathWorks, version 13), where spectral alignment was carried out, noisy regions were excluded, and data normalization was applied. Metabolite identification was performed using Chenomx^®^ software and further validated by comparison with the Human Metabolome Database (HMDB; www.hmdb.ca). The total peak area of each identified metabolite was annotated and integrated for subsequent statistical analysis.

### GPS procedures

During the matches, the players wore a porTable 10-Hz GPS unit (Playertek+; Catapult Innovations). This acquisition frequency has been shown adequate validity for detecting total distance covered in different velocity ranges, but with larger errors associated to very high speeds and accelerations and decelerations (Muñoz-López et al., 2017; Rampinini et al., 2014). After, the data were synchronized to a notebook (Catapult ONE Sync; Catapult) and analyzed using specific web cloud (Catapult One; Catapult Innovations), according to the manufacturer specifications. All athletes were evaluated either entire (with ∼90 min of durations) or part of the match (at least 25 min played) (Table 1). The GPS was started for at least 15 min before the athlete took the field. The player load, total distance covered (km), distance covered (m) in low speed (4–11 km/h), in high speed (11–18 km/h), in very high-speed (> 18 km/h and 18–25 km/h) and during sprints (> 25 km/h), maximum velocity (km/h), number of accelerations, decelerations, and number of impacts were the variables assessed during the thirteen matches included in the analysis.

**Table 1:** GPS variables for the 13 matches included in the analysis

	03.06	12.06	02.07	06.07	20.07	01.08	12.08	18.08	29.08	11.09	16.09	26.10	10.11	*P*-value
Number of players	14	10	10	12	11	11	12	10	12	11	15	10	13	*
Total Distance (km)	8.01 ± 0.53	8.63 ± 0.63	9.59 ± 0.63	9.47 ± 0.58	10.39 ± 0.60	10.27 ± 0.60	9.91 ± 0.58	9.90 ± 0.63	9.73 ± 0.58	9.37 ± 0.60	9.29 ± 0.52	9.69 ± 0.63	8.31 ± 0.55	0.13
Player load	363.63 ± 23.06	382.35 ± 27.28	429.84 ± 27.28	418.03 ± 24.91	476.71 ± 26.01	444.13 ± 26.01	431.92 ± 24.91	421.35 ± 27.28	424.68 ± 24.91	406.32 ± 26.01	407.78 ± 22.28	416.86 ± 27.28	372.21 ± 23.93	0.17
Dist 4–11 km/h (m)	2859.68 ± 201.03	2565.50 ± 237.86	2940.90 ± 237.86	2804.42 ± 217.13	3162.91 ± 226.79	2978.82 ± 226.79	2802.33 ± 217.13	2935.50 ± 237.86	2886.06 ± 217.13	2821.01 ± 226.79	2695.91 ± 194.21	2894.74 ± 237.86	2569.75 ± 208.62	0.90
Dist 11–18 km/h (m)	701.91 ± 63.90	666.60 ± 75.61	686.40 ± 75.61	740.58 ± 69.02	753.73 ± 72.09	819.27 ± 72.09	802.08 ± 69.02	786.90 ± 75.61	727.99 ± 69.02	719.55 ± 72.09	747.54 ± 61.73	772.77 ± 75.61	622.84 ± 66.31	0.78
Dist ≥ 18 km/h (m)	806.94 ± 95.09	747.47 ± 112.51	889.83 ± 112.51	862.83 ± 102.71	849.09 ± 107.28	1060.09 ± 107.28	1070.05 ± 102.71	883.71 ± 112.51	955.02 ± 102.71	862.19 ± 107.28	920.12 ± 91.87	991.84 ± 112.51	838.21 ± 98.68	0.64
Dist 18–25 km/h (m)	135.24 ± 15.98	107.00 ± 18.91	130.70 ± 18.91	127.17 ± 17.26	128.36 ± 18.03	152.27 ± 18.03	158.83 ± 17.26	128.90 ± 18.91	140.88 ± 17.26	128.44 ± 18.03	146.67 ± 15.44	146.78 ± 18.91	119.58 ± 16.58	0.58
Dist ≥ 25 km/h (m)	7.64 ± 5.38	17.40 ± 6.37	16.60 ± 6.37	16.75 ± 5.81	15.00 ± 6.07	23.64 ± 6.07	21.50 ± 5.81	18.60 ± 6.37	20.41 ± 5.81	16.91 ± 6.07	19.67 ± 5.20	20.66 ± 6.37	20.03 ± 5.59	0.77
Maximum Speed (km/h)	28.84 ± 0.63	30.23 ± 0.75	30.00 ± 0.75	30.22 ± 0.68	28.83 ± 0.71	31.79 ± 0.71	31.02 ± 0.68	31.09 ± 0.75	30.52 ± 0.68	30.14 ± 0.71	30.87 ± 0.61	30.87 ± 0.75	31.38 ± 0.66	0.78
Acc (n)	75.86 ± 5.53	67.80 ± 6.54	75.10 ± 6.54	78.92 ± 5.97	89.09 ± 6.24	78.36 ± 6.24	80.25 ± 5.97	78.50 ± 6.54	74.17 ± 5.97	83.82 ± 6.24	81.33 ± 5.34	81.10 ± 6.54	75.62 ± 5.74	0.79
Decc (n)	74.50 ± 6.26	73.60 ± 7.40	83.40 ± 7.40	87.83 ± 6.76	96.09 ± 7.06	89.45 ± 7.06	87.00 ± 6.76	87.90 ± 7.40	82.25 ± 6.76	82.18 ± 7.06	86.40 ± 6.04	91.20 ± 7.40	79.77 ± 6.49	0.62
Impacts (n)	2.93 ± 2.19	2.80 ± 2.59	4.30 ± 2.59	3.50 ± 2.37	11.82 ± 2.47	3.09 ± 2.47	3.08 ± 2.37	3.40 ± 2.59	4.00 ± 2.37	4.36 ± 2.47	2.93 ± 2.12	3.80 ± 2.59	5.15 ± 2.27	0.56

### Statistical analysis

Characterization of the participants was presented as mean values and ± standard deviation (SD). To observe potential candidate metabolites, heat map was generated with the concentrations of metabolites for all players. For the analysis of the 20 cross-sectional points across the season, Principal component analysis (PCA) was conducted to identify possible outliers among all data (See Supplementary Figure – S1). Orthogonal partial least square – Discriminant analysis (OPLS-DA) was performed to verify separation between early (first ten matches) and late (last ten matches) season. Also, for those data, a heat map was generated applying hierarchical cluster analysis (HCA) only in the y-axis, to preserve chronological order along the season and interpret metabolic shifts over time. The heat map reports an increase or decrease in each metabolite in relative concentration as a red color and a blue color, respectively. Heat map analysis was also employed for the thirteen matches with GPS data matching urine sampling. In this case, HCA was used in both axis, metabolites and matches. GPS-based variables were included in this heatmap but were not included in the hierarchical clustering analysis due to differences in data dimensionality and scale relative to metabolomic variables. Including both datasets in a single clustering procedure could bias the distance calculations, leading to dominance of the higher-dimensional GPS data and obscuring metabolite-driven patterns. Therefore, clustering was performed using metabolomic data only. For the GPS data, normality was tested using Kolmogorov-Smirnov’s test, and Homogeneity was tested using Levene’s test. Depending on if the assumptions were met, one-way ANOVA or Kruskal-Wallis test for independent groups were calculated to compare GPS variables among matches. A Pearson’s correlation matrix was calculated between GPS-derived variables and metabolites. Statistical significance was adopted as *p* < 0.05.

## Results

GPS variables were compared among the thirteen matches included in the analysis (Table 1). Total distance covered and Player Load were the variables with the most variability throughout matches along the season. However, there were no GPS variables with statistically significant differences along the matches analyzed throughout the period of this study. This suggests a lack of change in the external load of the group along the season. The statistical analysis was performed on independent groups, because of substitutions, injury or the trade of players along the season. So, if there is any change in the individual load of the matches along the season, the GPS group analysis is not capable to show statistically significant differences.

**Fig. 1:**
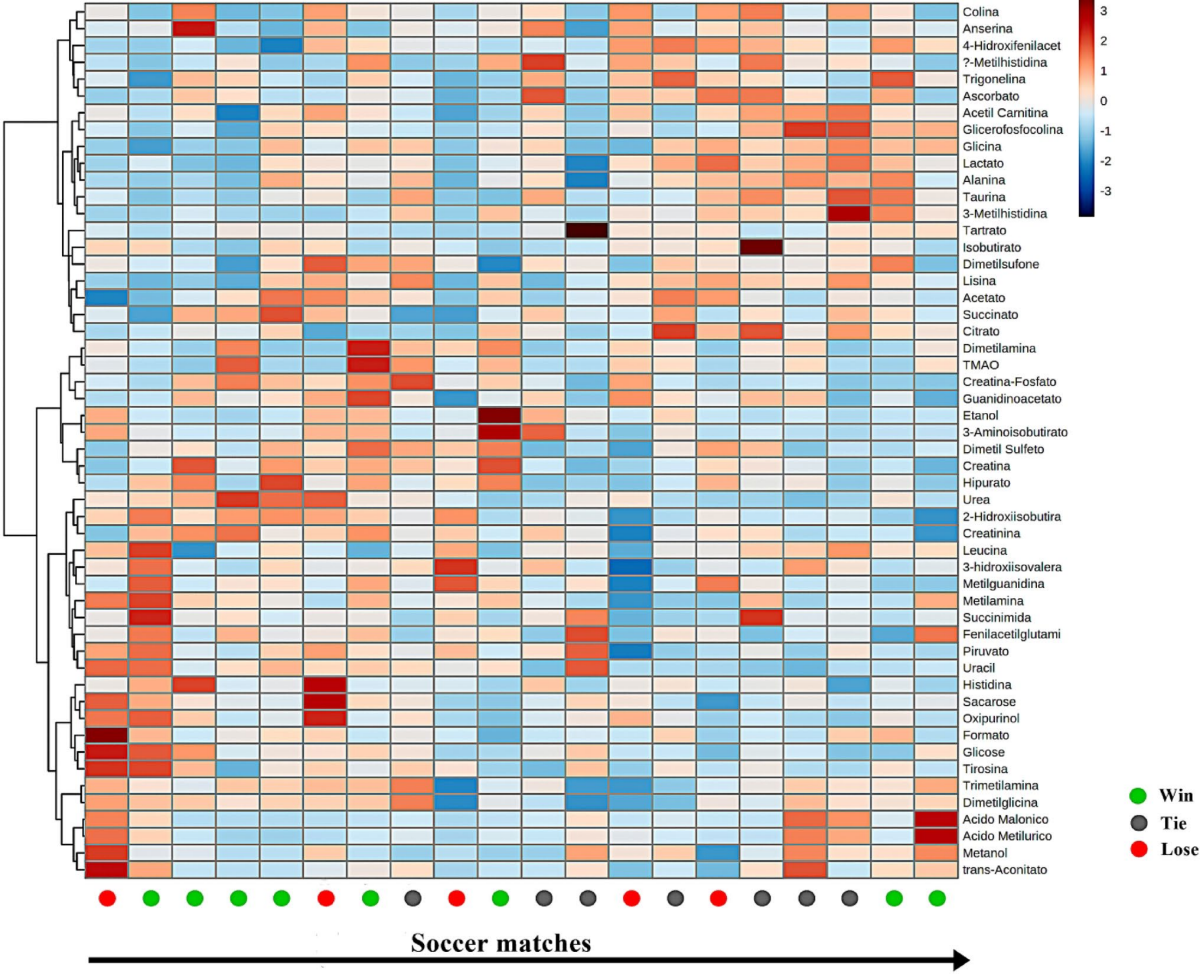
The heatmap displays changes in each metabolite, with red representing increases and blue reporting a decrease in metabolites. The metabolites are listed at the left side of each row, and the matches are shown at the bottom of each column. Hierarchical cluster analysis was performed only in the Y-axis (metabolites)

During the studied period, the team won 60% of the first ten soccer matches with only one draw and three losses. On the other hand, the last ten soccer matches involved only two wins. The heat map (Fig. 1) showed two bigger clusters: #1 from Choline to Citrate and #2 from Dimethylamine to trans-Aconitate. It is possible to verify a trend where there are lower metabolite concentrations of this cluster at the beginning of the season, with an overall increase at the end of the season, while the second cluster behaves in general on the other way around. An exception to this trend would be a smaller cluster inside the second big cluster – between Trimethylamine and trans-Aconitate. This sub-cluster shows a three-phase trajectory, being overall higher at the start of the season, getting lower in the middle and then increasing again at the end of the season.

**Fig. 2:**
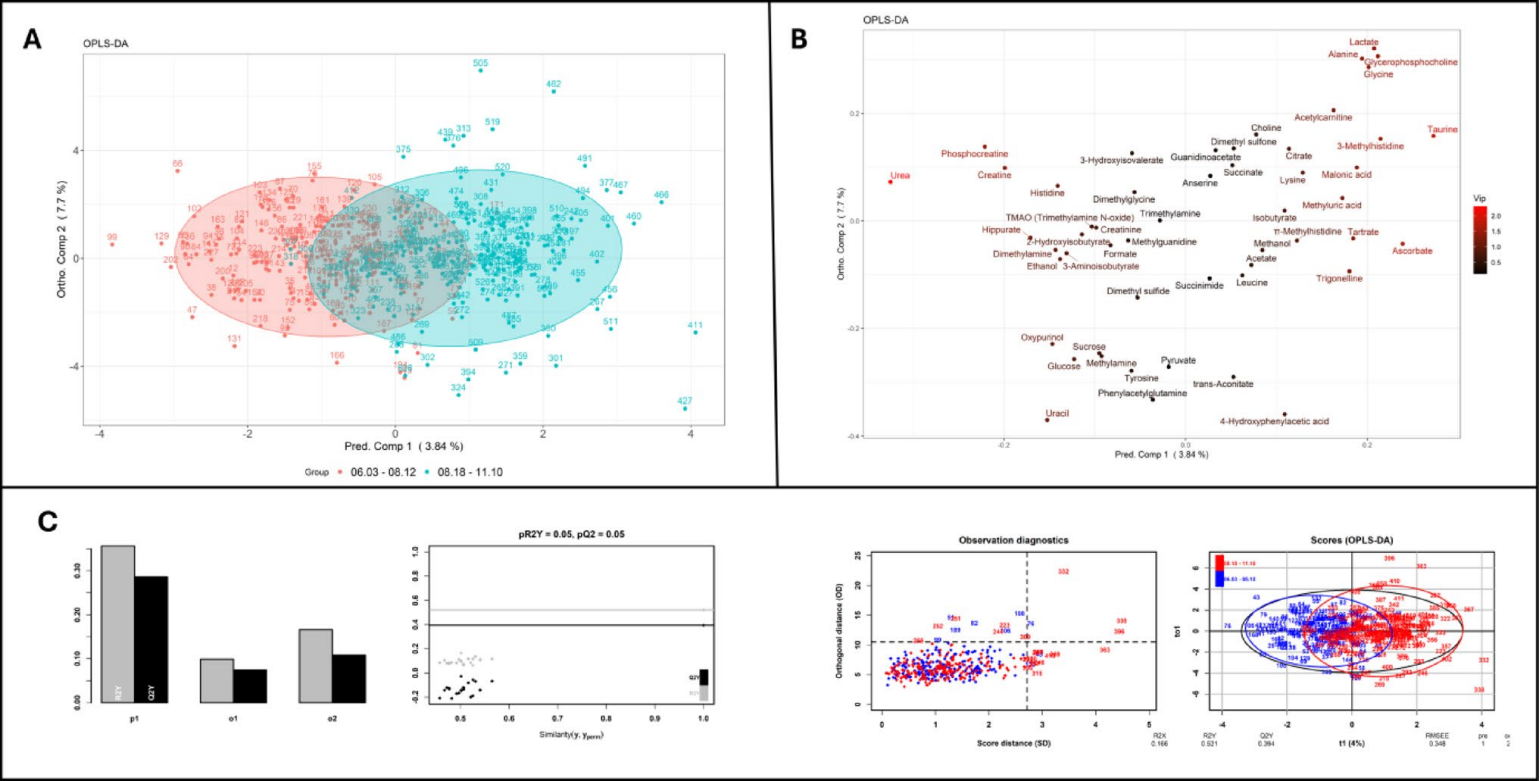
OPLS-DA analysis comparing samples from the first ten (red) and the last ten (blue) matches in the season. Panel **A** shows the scores, panel **B** the loadings with vip scores and panel **C** the quality of the model

To follow the trend perceived in Fig. 1, a PCA analysis (See Supplementary Material, Figure - S1), followed by OPLS-DA analysis (Fig. 2) showed a considerable amount of separation between two groups, the first and last ten matches analyzed. This supports the notion of a change in the metabolomic profile from the star to the end of the season.

**Fig. 3:**
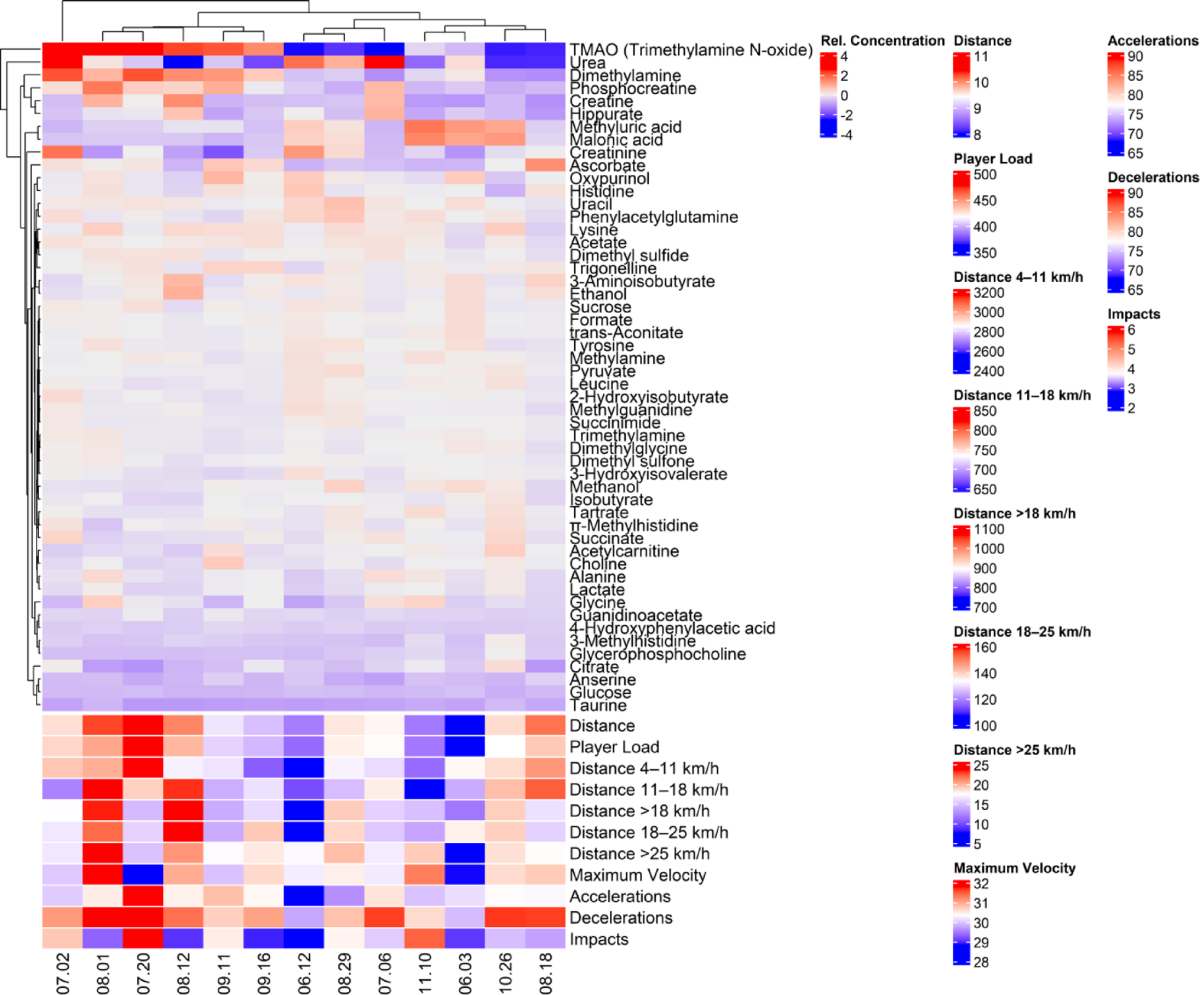
Heat map of the metabolites and GPS-derived variables. Hierarchical cluster analysis was performed considering only the metabolites, disregarding GPS variables. GPS variables were added in order to show their behavior along the clustering of metabolites

Using a subset of the matches (13 games) to compare the metabolomics results to the GPS data, it is possible to see in Fig. 3 two vertical clusters, being the first one between matches 08.01 and 09.16, and the second one between matches 06.12 and 08.18. Despite the individual fluctuations, variables of covered distance in different speed zones tended to be higher in the first cluster and lower in the second cluster, overall. The clustering of metabolites (y-axis) was sparce and difficult to describe. Even so, the fact that the HCA based on pre-match metabolites state was able to create a visible pattern of the GPS variables reinforce the shared variance of the external and internal match load when measured by GPS and metabolomics.

**Fig. 4:**
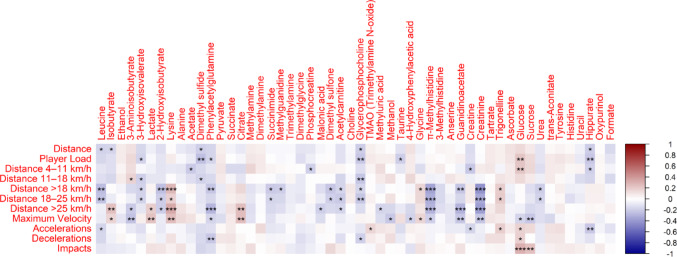
Matrix of paired Pearson’s correlations between GPS-derived variables and metabolites. Red means positive correlations, blue means negative correlations. * - *P* < 0.05;, ** - *P* < 0.01; *** - *P* < 0.001

Furthermore, GPS and metabolites showed significant correlations (Fig. 4). For example, we found inverse correlations amongst Leucine, Guanidinoacetate, Creatinine, and 3-Hydroxyisovalerate with several variables of covered distance in different speed zones. Considering these metabolites link to nitrogen metabolism and protein catabolism, a higher value on those markers before the match indicated lower covered distances in high and low intensities. This way, players with the most amount of muscle damage and stress markers may have been experiencing an incomplete recovery that may have affected their ability to perform. Similarly, the significant inverse correlation among Glycerophosphocholine (GPC) to most variables of covered distance in different speed zones, except for high intensity ones (direct but not significant), indicates this may be an interesting marker to correlate muscle integrity and the readiness to perform higher values of external load during soccer matches. Higer values of GPC indicate lower cell integrity and turnover in cancer patients (Sonkar et al., 2019), and those players with lower values of GPC also were prone to perform better based on this correlation. Future studies may focus on causality of those correlations, since evidence of GPC use as a biomarker outside cancer patients is scarce.

Finally, there were direct correlations for Glucose and player load, accelerations, decelerations and impacts, as well as for Citrate to both Distance > 25 km/h and Maximum Velocity. This indicates that the high availability of glucose, as well as high TCA activity pre-match are related to high intensity efforts.

## Discussion

While few studies investigated metabolomic changes throughout the soccer season at specific time points (Pintus et al., 2020; Quintas et al., 2020), evaluating metabolomic alterations match by match along the season remains unexplored. The current study is the first to investigate the alterations in metabolomic profiling by observing several cross-sectional moments – close to official matches – along an entire season in a professional Brazilian team. Metabolomics analyses showed that at rest (first urine of the day), some metabolites related to muscle energy metabolism (Citrate, Succinate, Lactate, Alanine) and protective pathways (Taurine, Ascorbate) were lower at the start of the season, when 60% of the studied outcomes were wins (the first ten soccer matches). On the other hand, those metabolites were higher at the end of the season, which may indicate an inadequate time for recovery of those energetic pathways. However, some metabolites related to muscle damage (Urea, Creatinine, 3-Hydroxyisovalerate), alactic energy metabolism (Creatine and Phosphocreatine) as well as to fatigue (Guanidinoacetate – diminishes with the occurrence of fatigue) were higher at rest in the early season, and tended to decrease in the last ten soccer matches (only 20% of wins). Even if these responses are referred to a correlation matrix, fluctuations in metabolites should be interpreted cautiously about metabolic pathways.

Earlier in the season, the correlation matrix through heatmap assessed via 1 H-NMR (Fig. 1) showed lower concentrations of Citrate, Succinate, Lactate, Alanine that increased over time. All those metabolites are related to the TCA cycle and glycolysis, indicating a lower activity of those pathways at the beginning of the season. These metabolites are mainly related to high-intensity activity performed during the match. High-intensity activities (i.e., accelerations, jumping, sprints, etc.) are decisive aspects of a soccer match and contribute to a win (Bangsbo et al., 2007; StØlen et al., 2005). In addition, there is evidence showing that match result (Bradley & Noakes, 2013; Chmura et al., 2018) and opponent (Rampinini et al., 2007) modulates high-intensity activities. It is possible to speculate that the last matches increased the need for utilization of energy pathways due to the increased difficulty of the matches – which was not possible to show only using GPS-derived variables. The drop in winning percentage (i.e., 60% wins vs. 20% wins) are in line with this hypothesis. Thus, the presence of these metabolites may represent a more intense performance at the end of the season. In the same manner, the first cluster shows metabolites linked to mitochondrial function (Acetylcarnitine - marker of fatty acid oxidation capacity) (McCann et al., 2021), and antioxidant function (Taurine) (Surai et al., 2021) increasing over time at rest, indicating a possible mitochondrial strain, accumulated oxidative stress and reduced recovery efficiency towards the end of the season.

The second cluster showed a decreasing profile over the season for resting creatine and phosphocreatine concentrations in the urine, which supports a higher availability of those metabolites from the anaerobic alactic metabolism (Hargreaves & Spriet, 2020) at the start of the season. This is in line with other metabolites (i.e., formate and pyruvate) associated with anaerobic metabolism (Jang et al., 2018), were also elevated in the first portion of the season. During a soccer match, decisive actions are performed at high intensity, promoting a more significant disturbance of the internal environment in the skeletal muscle (Kakavas et al., 2021); high levels of metabolites may represent a high number of decisive offensive actions, representing a positive factor in maintaining performance. The latter decrease of those metabolites along the season may indicate a less activity of the anaerobic alactic metabolism, thus lower number of decisive actions. The match status (e.g. winning, drawing or losing) is known to affect the intensity of the match (Moalla et al., 2018). Considering there were more wins at the start of the season, the energetic pathways may have shifted from a higher anaerobic alactic metabolism (decisive actions resulting in goals) to enhance the use of the TCA cycle (Citrate, Succinate) and lactic glycolysis (Lactate) (defensive actions and incapacity to create goal opportunities).

In a similar way, the reduction of Guanidinoacetate post-exercise-induced fatigue (Al Fazazi et al., 2019; Stajer et al., 2016). The reduction of this metabolite along the season may represent an accumulation of fatigue, since Guanidinoacetate is a precursor of Creatine (Ostojic et al., 2020), which is also diminishing throughout the season. The lower availability of Creatine at rest may explain the need for a shift in the energetic pathway predominance, influencing the capability to perform at higher intensities, reduced levels of muscle damage biomarkers at the end of the season.

Those energetic shifts from alactic anaerobic metabolism to a higher use of glycolysis and the TCA cycle along the season are in line with the early higher values of Urea, Creatinine, 3-Hydroxyisovalerate. Those metabolites are linked to a higher amount of muscle damage, protein catabolism, and nitrogen metabolism. Other metabolites allocated in the second cluster were Hippurate and Dimethylglycine, which are related to muscle damage and cell disturbance (Neal et al., 2013). The presence of these metabolites often occurs after constant accelerations and decelerations (Jang et al., 2018) or high-intensity exercises (Neal et al., 2013), which may be associated with muscle damage. However, our data could not confirm a direct association between the enhancement of those metabolites and a greater number of accelerations and decelerations, since no significant differences were found between GPS variables across the studied period. This pattern is not in line with the body of evidence, since previous studies have shown greater high-intensity running and distance covered at the end of the season compared to the beginning and middle of the season (Rampinini et al., 2007; João R Silva et al., 2013). This increase in GPS-provided game performance variables may be related to the season’s key moments (i.e., final matches and playoffs) (Rampinini et al., 2007; João R Silva et al., 2013). The fatigue profile indicated by the metabolomics results may explain the absence of enhancement in the GPS variables. It is possible that other teams suffering less from fatigue were able to increase their external load efforts from the mid to late season, explaining the diminished rate of winning for the studied team during the last games.

It is important to state that the absence of significant differences for the GPS variables may be due to the use of independent groups hypothesis testing, which may not have been sensitive to small but important individual changes across the games. However, other plausible explanation lies in the magnitude of error of GPS systems, which tend to be bigger for short distances and high intensity efforts, especially when involving changes of direction (Beato et al., 2016; Muñoz-López et al., 2017; Rampinini et al., 2014; Varley et al., 2012). Our results indicate that the GPS system may be missing important internal load stress throughout the season. Considering coaches often rely on GPS systems to adjust training loads (Ehrmann et al., 2016; Ravé et al., 2020), the inclusion of approaches more sensitive to homeostatic disturbances – such as metabolomics – may improve the training load adjustment for soccer players.

Despite our GPS analysis not showing differences among the games throughout the season (Table 1), Fig. 3 shows a pattern of accommodation of the metabolites’ response and the GPS pattern across the matches studied. It is crucial to understand that, unlike Fig. 1 where the chronological order was preserved, the clustering in Fig. 3 was based on both the x-axis (matches) and y-axis (metabolites). The GPS variables were displayed only for qualitative interpretation of the relationships between metabolites and GPS outcomes but were not included for the hierarchical clustering analysis. Even so, the clustering of the metabolites could show a pattern in the GPS variables, tending to be higher in the first vertical cluster (between games 07.02 to 09.16) and lower in the second vertical cluster (between games 06.12 to 08.18). This indicates that GPS and metabolites from NMR metabolomics share variation, and possible cause and effect relationships must be further studied.

The negative association between urinary metabolites related to branched-chain amino acid catabolism and nitrogen metabolism (e.g., Leucine, 3-hydroxyisovalerate, Creatinine, Guanidinoacetate) and GPS-derived performance metrics suggest lower accumulated protein catabolism is linked to greater external load during matches. Given that urine samples were collected prior to competition, a high value on these metabolites likely reflects accumulated physiological stress and incomplete recovery from prior efforts, rather than acute responses to the match itself. The evidence of serum, plasma and saliva biomarkers of muscle damage as a result of soccer training and matches is not uncommon (Csala et al. 2021; Thorpe and Sunderland 2012b). However, a systemic, omics approach investigating the accumulation of a broader number of biomarkers, with this much time points across a soccer season is the novelty of this investigation. It is essential to recognize some limitations. Data regarding nutrition, sleep control, and training load were not reported. However, all athletes were sub-elite soccer team professionals who had routine orientation, dietary planning and training control by their club. We cannot rule out those aspects as possible confounder factors, but because the club control, it is safe to assume this is a issue in a less controlled cohort of participants. This study was descriptive, limited to statistical correlations and trends of change across the season, but it provided a proof of concept for the use of metabolomics to complement GPS data as tools for monitoring internal and external loads in soccer. Future investigative studies will be conducted to explore the dose-response relationship between physical effort and metabolites using this information and target analysis.

In addition, GPS data were analyzed only during soccer matches, and training sessions were not provided for publication by the coaching staff. It would be of paramount importance to have the training sessions GPS variables, but we are assuming the level of effort from the training process is more stimulated during the match itself, where the player must perform at his peak to win. Additionally, the soccer season calendar is full, with two to three matches per week, and as consequence more substantial efforts are made during the matches. Also, it is essential to note that research with professional soccer players during competitive season is a challenging process. Therefore, the limitations presented here are counterbalanced by the high ecological validity of the experimental design, which followed the routine of an actual professional team along an entire season. Future studies must use this proof of concept to investigate cause and effect of the predictive power of metabolomics for fatigue and muscle damage.

## Conclusion

In summary, our data showed that metabolites associated to muscle energy metabolism and protective pathways (i.e. Phosphocreatine, Creatine, Citrate, Succinate, Lactate, Alanine, Taurine, Ascorbate) were associated with a better success period of the season (6 wins, 1 draw, 3 losses). On the other hand, resting profile in the last part of the season (2 wins, 6 draws, 2 losses) presented lower values of metabolites linked to muscle damage (Urea, Creatinine, 3-Hydroxyisovalerate), and higher indication of fatigue (Guanidinoacetate). The correlations between some of those biomarkers to GPS-derived variables is a proof of concept that metabolomics can be used as a complementary tool for assessing the performance readiness in male soccer players regarding fatigue, muscle damage and bioenergetic pathways.

## Supplementary Information

Below is the link to the electronic supplementary material.

Supplementary material 1 (DOCX 270.5 kb)

## Data Availability

No datasets were generated or analysed during the current study.
