# Supplementary material for: NMR-based metabolomics profile during a soccer season of a sub-elite soccer team
Source: Metabolomics. 2026 May 24;22(3):86. doi: 10.1007/s11306-026-02452-2 (PMC13199214; doi:10.1007/s11306-026-02452-2)
Supplement: Supplementary file 1 — Supplementary material 1 (DOCX 270.5 kb) [file 11306_2026_2452_MOESM1_ESM.docx]

**Supplementary material**

**Title:** NMR-based metabolomics profile during a soccer season of a sub-elite soccer team

Supplementary information for the analysis of the 20 cross-sectional time points across the season, principal component analysis (PCA) was performed to identify potential outliers, as well as underlying patterns, trends, and discriminant metabolites within the dataset.

**
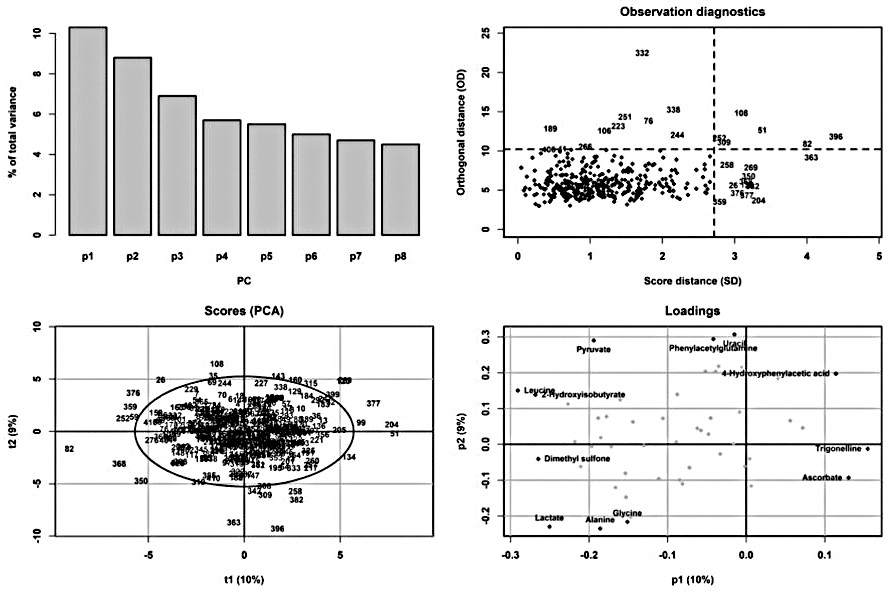
**

**Figure S1-A.** *Principal Component Analysis (PCA) overview including explained variance (scree plot), observation diagnostics (score vs orthogonal distance), scores plot, and loadings plot.*

**
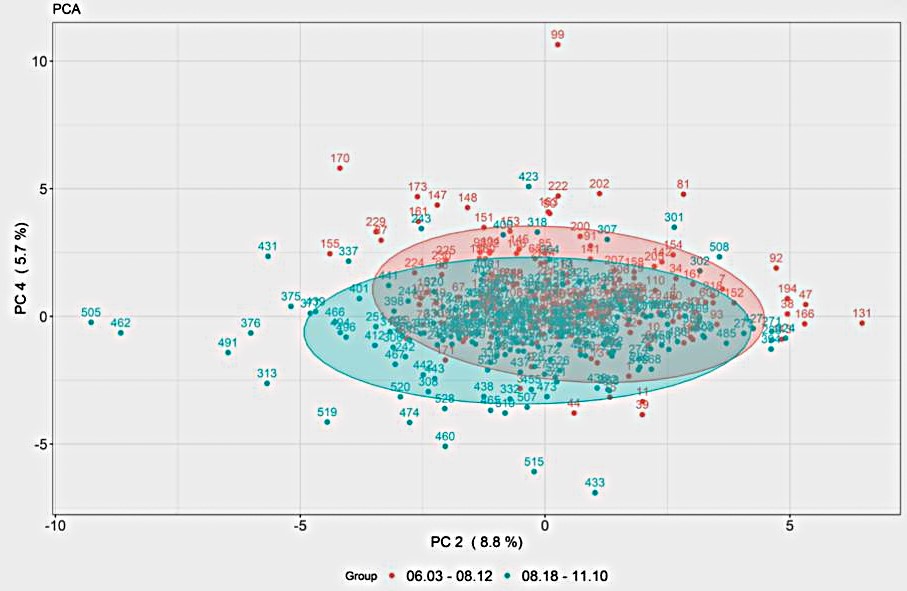
**

**Figure S1-B.** *PCA scores plot showing group distribution across sampling periods (between 06.03-08.12 period (reg group) vs between 08.18-11.10 period (blue group) with confidence ellipses.*

**
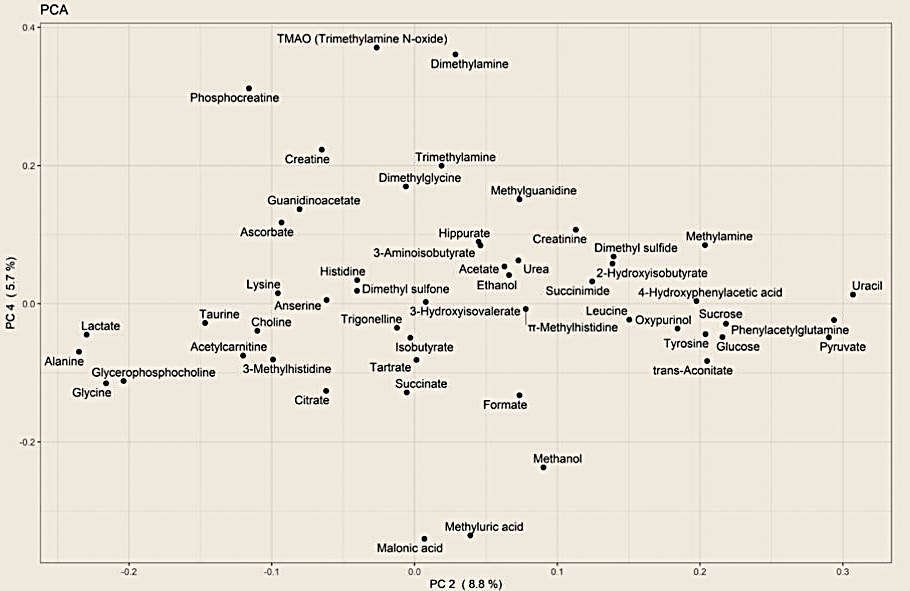
**

**Figure S1-C.** *PCA loadings plot highlighting metabolite contributions to principal components and metabolic profile separation.*
